# Archaeal nucleosome positioning in vivo and in vitro is directed by primary sequence motifs

**DOI:** 10.1186/1471-2164-14-391

**Published:** 2013-06-10

**Authors:** Narasimharao Nalabothula, Liqun Xi, Sucharita Bhattacharyya, Jonathan Widom, Ji-Ping Wang, John N Reeve, Thomas J Santangelo, Yvonne N Fondufe-Mittendorf

**Affiliations:** 1 Department of Molecular and Cellular Biochemistry, College of Medicine, University of Kentucky, Lexington, KY 40536, USA; 2 Department of Statistics, Northwestern University, Chicago, IL 60208, USA; 3 Department of Molecular Biosciences, Northwestern University, Evanston, IL 60208, USA; 4 Department of Microbiology, Ohio State University, Columbus, OH 43210, USA; 5 Center for RNA Biology, Ohio State University, Columbus, OH 43210, USA

**Keywords:** Archaea, Nucleosome positioning, Dinucleotide repeats, Histone deletions, rDNA expression, Chromatin evolution

## Abstract

**Background:**

Histone wrapping of DNA into nucleosomes almost certainly evolved in the Archaea, and predates Eukaryotes. In Eukaryotes, nucleosome positioning plays a central role in regulating gene expression and is directed by primary sequence motifs that together form a nucleosome positioning code. The experiments reported were undertaken to determine if archaeal histone assembly conforms to the nucleosome positioning code.

**Results:**

Eukaryotic nucleosome positioning is favored and directed by phased helical repeats of AA/TT/AT/TA and CC/GG/CG/GC dinucleotides, and disfavored by longer AT-rich oligonucleotides. Deep sequencing of genomic DNA protected from micrococcal nuclease digestion by assembly into archaeal nucleosomes has established that archaeal nucleosome assembly is also directed and positioned by these sequence motifs, both in vivo in *Methanothermobacter thermautotrophicus* and *Thermococcus kodakarensis* and in vitro in reaction mixtures containing only one purified archaeal histone and genomic DNA. Archaeal nucleosomes assembled at the same locations in vivo and in vitro, with much reduced assembly immediately upstream of open reading frames and throughout the ribosomal rDNA operons. Providing further support for a common positioning code, archaeal histones assembled into nucleosomes on eukaryotic DNA and eukaryotic histones into nucleosomes on archaeal DNA at the same locations. *T. kodakarensis* has two histones, designated HTkA and HTkB, and strains with either but not both histones deleted grow normally but do exhibit transcriptome differences. Comparisons of the archaeal nucleosome profiles in the intergenic regions immediately upstream of genes that exhibited increased or decreased transcription in the absence of HTkA or HTkB revealed substantial differences but no consistent pattern of changes that would correlate directly with archaeal nucleosome positioning inhibiting or stimulating transcription.

**Conclusions:**

The results obtained establish that an archaeal histone and a genome sequence together are sufficient to determine where archaeal nucleosomes preferentially assemble and where they avoid assembly. We confirm that the same nucleosome positioning code operates in Archaea as in Eukaryotes and presumably therefore evolved with the histone-fold mechanism of DNA binding and compaction early in the archaeal lineage, before the divergence of Eukaryotes.

## Background

Histone wrapping of nuclear DNA generates nucleosomes, the basic unit of chromatin in virtually all Eukaryotes. Nucleosomes are dynamically associated with the genome and their distribution is not random, but often plays a major role in regulating gene expression [1-4]. Nucleosome assembly is favored or deterred by differences in the enthalpic and entropic costs inherent in wrapping and maintaining different sequences in the rigid nucleosome toroid, and this has resulted in a eukaryotic nucleosome positioning code [1,3,5-8]. Histones, and presumably histone-DNA interactions, evolved before the divergence of the archaeal and eukaryotic lineages with histones now distributed throughout the *Euryarchaea*, *Nanoarchaea* and *Thaumarchaea* and also present in some *Crenarchaea*[9-11].

Sequencing DNA molecules selected from a large random population by repetitive selection (SELEX) and PCR-amplification, based on increased archaeal histone-DNA affinity, revealed that DNA molecules preferentially assembled into archaeal nucleosomes in vitro had sequences that conformed to the eukaryotic nucleosome positioning code [12]. However, the technology was not then available to determine if this was also the case in vivo and so confirm in vivo these results obtained in vitro. But, with the advent of large-scale DNA sequencing technologies, such comparisons became possible, and here we document that archaeal nucleosome assembly in vivo is directed by the nucleosome positioning code in both *Methanothermobacter thermautotrophicus* and *Thermococcus kodakarensis*, and that this is reproduced in vitro with archaeal genomic DNA and purified archaeal or eukaryotic histones. With recently developed genetic technologies, it was possible to delete either, but not both of the two archaeal histone-encoding genes present in *T. kodakarensis* generating strains that grow normally but exhibit transcriptome changes when compared with the parental strain [13]. Here we show that the histone deletions also result in changes in the archaeal nucleosome profiles upstream of the genes that have increased or decreased transcription.

## Results

### Nucleosome positioning motifs in vivo

**Figure 1:**
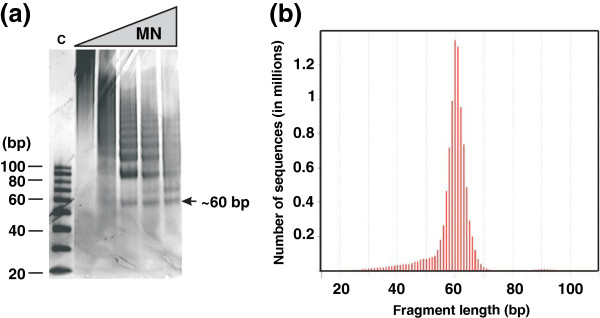
**Archaeal nucleosomes protect ~60 bp chromosomal DNA fragments from micrococcal nuclease (MN) digestion. (a).** Ethidium bromide stained electrophoretic separation of DNA molecules protected from MN digestion of *T. kodakarensis* TS517 chromatin. The control lane (C) contained double-stranded DNA size standards (bp). As indicated, MN digestion generated DNA molecules that migrated to form a band consistent with ~60 bp molecules. These were isolated and sequenced. **(b).** The number and size profile of the sequences of the ~60 bp DNA fragment generated by ABI-SoLiD deep sequencing.

**Figure 2:**
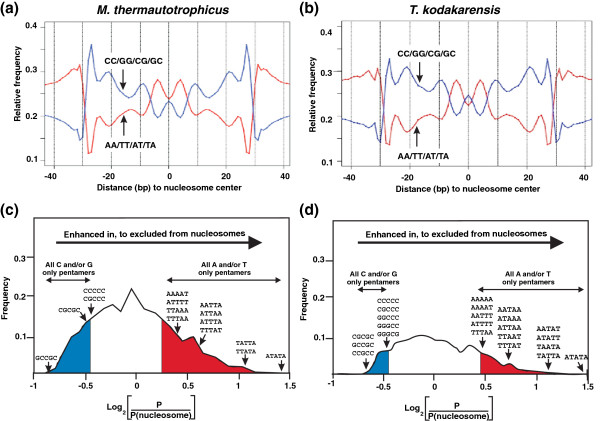
**Archaeal nucleosomes assembled *in vivo *contain offset helical repeats of AA/AT/TA/TT and CC/GG/GC/CG dinucleotides and lack oligo A/T-rich sequences.** The frequencies of occurrence of AA/AT/TA/TT (red line) and CC/GG/GC/CG (blue line) dinucleotides, at each position relative to the center of archaeal nucleosomes assembled (**a)** in *M. thermautotrophicus* and **(b)** in *T. kodakarensis* (**c** and **d**). The ratios of the presence and absence of all pentamers in the DNA molecules protected from MN digestion by nucleosome assembly in *M. thermoautotrophicus* and in *T. kodakarensis*, respectively. The graphs show the ratio of occurrence of each of the 1024 possible pentamers in nucleosomal DNA (P_nucleosome_) versus in non-nucleosomal DNA (P). As examples, the specific locations on the curves of representative G and/or C only, and A and/or T only pentamers are indicated. As noted, all 32 G and/or C-only pentamers were located preferentially within nucleosomal DNA (blue shaded region), whereas all 32 A and/or T-only pentamers were preferentially excluded from nucleosome incorporation (red shaded region).

The discovery of archaeal histones [14] and most subsequent studies [9,15], have investigated histones from methanogens for which genetic procedures are not available. We therefore first determined, and confirmed, that archaeal nucleosome positioning in vivo was directed by same sequence motifs in *M. thermautotrophicus* and *T. kodakarensis* and then focused on *T. kodakarensis,* as the experimental system, for which genetic procedures and strains with histone genes deleted were available [13]. As previously documented for *M. thermautotrophicus*[16], MN digestion of chromatin from *T. kodakarensis* generated a ladder of discrete-length DNA molecules, with a predominant population of ~90 bp molecule first accumulating, consistent with the length of a DNA molecule required to completely encircle an archaeal histone tetramer core [17,18]. With further MN digestion, the number of ~90 bp molecules decreased and ~60 bp molecules accumulated (Figure 1a), the length of DNA directly bound by the histone folds of a histone tetramer. Deep-sequencing of the ~60 bp fragments resulted in 7 and 9 million unique reads, respectively, of molecules with lengths ranging from 55 to 65 nucleotides from *M. thermautotrophicus* and *T. kodakarensis* (Figure 1b). As established for DNA molecules preferentially packaged into eukaryotic nucleosomes [1,3,5-8,19-21], these sequences were dominated by helical repeats (10 bp) of AA/AT/TA/TT dinucleotides offset by 5 bp from repeats of GG/GC/CG/CC dinucleotides with the centers preferentially filled by GC-rich sequences (Figure 2a and 2b).

In contrast to A/T-rich dinucleotides that, in a 10 bp periodicity, offer flexibility and so facilitate DNA incorporation, poly (dA:dT) tracts are relatively rigid. This deters their incorporation into nucleosomes [1-3,6,8,22,23] and poly (dA:dT) tracts are significantly underrepresented in DNA incorporated into eukaryotic nucleosomes [1,6-8,23-33]. Analyses of the archaeal ~60 bp nucleosomal DNA fragments revealed that A/T-rich oligonucleotides were also excluded from incorporation into archaeal nucleosomes assembled in *M. thermautotrophicus* and *T. kodakarensis*. For example, as illustrated in Figures 2c and 2d, when the frequencies of occurrence of each of the 1064 pentamers was determined, all of the 32 pentamers that contain only A and/or T were underrepresented relative to the presence in the genome sequences with ATATA (=TATAT) being the most disfavored pentamer in both *Archaea*. Oligo A/T-rich sequences are consequently located preferentially in nucleosome-depleted regions. In contrast, all of the 32 pentamers that contain only G and/or C were enriched in the ~60 bp MN-protected fragments generated from both *Archaea* relative to their abundances in the genome sequences (Figure 2c and 2d).

### Exclusion of nucleosomes from intergenic regions

**Figure 3:**
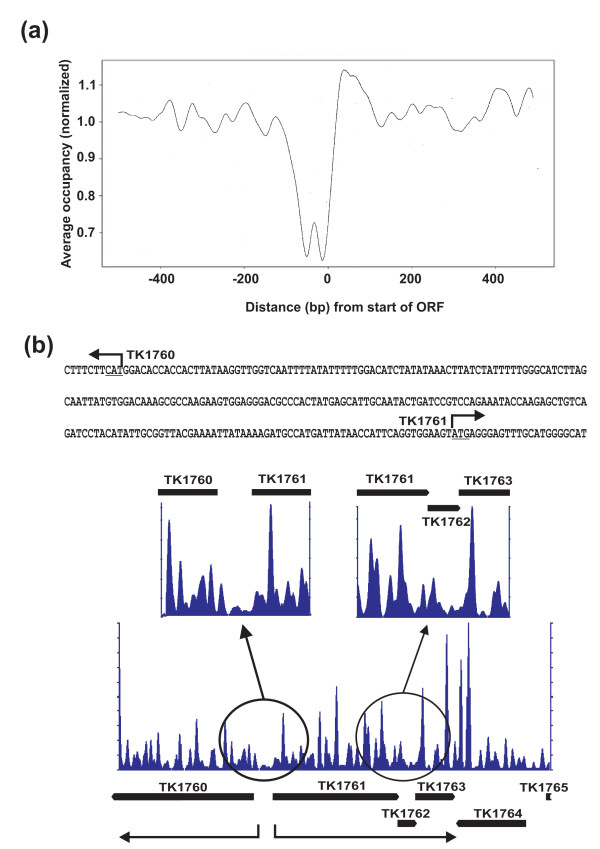
**Depletion of archaeal nucleosomes in intergenic regions. (a)**. The occurrence of nucleosomes relative to the start codons of all open reading frames (ORFs), as documented in the Archaeal Genome Browser [39]. The frequency of occurrence of each nucleosome read is plotted relative to the average value of occurrence of all nucleosomal reads sequenced from the *T. kodakarensis* TS517 genome. **(b)** The sequence of the intergenic region separating TK1760 and TK1761 is positioned above the profiles of nucleosomes assembled in vivo at this locus and downstream, within the well-established TK1761-TK1762-TK1763 operon [40].

In *T. kodakarensis*, at least 92% of the genome is coding sequence [34], and most intergenic regions are <100 bp, with many <50 bp. Within these regions, both transcription initiation (TATA-box sequences) and termination (oligo A/T-rich sequences) are directed by A/T-rich sequences [35,36]. Given compliance with the nucleosome positioning code [5-8,19,37,38], archaeal nucleosome assembly should avoid intergenic regions and this was confirmed by aligning the ~60 bp nucleosomal sequences with the genome sequence. A transcriptome map has not been established for *T. kodakarensis* but, based on bioinformatic predictions [39], non-transcribed intergenic regions are preferentially depleted of nucleosomes. Genome-wide, there was a substantial underrepresentation of nucleosomes immediately upstream of translation initiation codons (Figure 3a) and, in multigene operons, this was predominantly upstream of the promoter proximal gene. For example, TK1761-TK1762-TK1763 constitutes an operon transcribed divergently from TK1760 [40]. The intergenic region separating TK1760 and TK1761 contains several oligo A/T-rich sequences and had minimal nucleosome occupancy in vivo (Figure 3b). There was no similar region of nucleosome exclusion downstream from the promoter within the TK1761-1763 operon.

### Conservation of archaeal nucleosome positioning in vivo and in vitro

**Figure 4:**
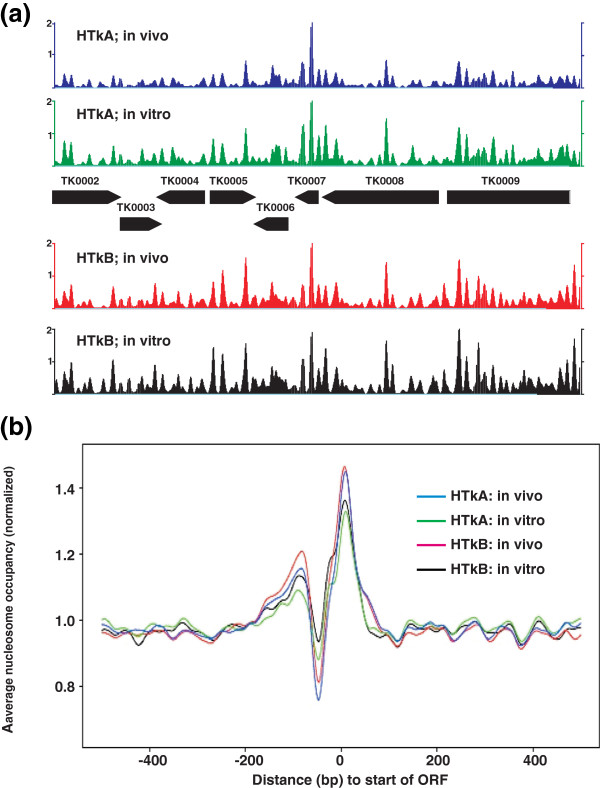
**Archaeal nucleosome profiles in vivo are reproduced in vitro. (a)** Profiles of the archaeal nucleosomes assembled in vivo and in vitro by HTkA and HTkB between nucleotide positions 5,000 and 10,000 on the *T. kodakarensis* genome. The organization of *T. kodakarensis* genes in this region is shown between upper and lower panels [34,39,42]. **(b)**. The occurrence of nucleosome positions relative to the start codon of all open reading frames (ORFs), assembled by HTkA in vivo (blue line) and in vitro (green line), and by HTkB in vivo (red line) and in vitro (black line) normalized to the total number of nucleosomal reads from each sequencing experiment.

**Figure 5:**
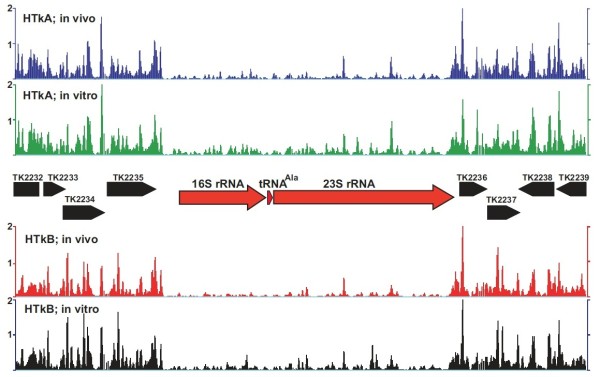
**Archaeal nucleosomes do not assemble on the ribosomal DNA operon.** Profiles of the archaeal nucleosomes assembled by HTkA and HTkB in vivo in *T. kodakarensis* LC125 and LC124, respectively, and in vitro on a 10 Kbp region of the *T. kodakarensis* genome. As illustrated, this region encodes the 16S and 23S rRNA (rDNA operon; red arrows) and several protein-encoding flanking genes [34,39].

Most archaeal histones are single histone folds that form homodimers in solution [9]. However, when mixed with other archaeal histone homodimers, there is rapid and spontaneous reassembly that generates an equilibrium mixture of the homodimers and all possible heterodimers [18]. *M. thermautotrophicus* has three histones [41] and *T. kodakarensis* has two histones [34] and, practically, it is impossible to know their homo- versus heterodimer configurations in vivo, a concern that limits the reproducibility in vitro of results obtained in vivo. The two histones in *T. kodakarensis*, HTkA and HTkB are encoded by TK1413 and TK2289, respectively, and although constructing a strain with both genes deleted proved impossible, *T. kodakarensis* LC124 (ΔTK1413) and LC125 (ΔTK2289) were constructed [13]. These strains therefore contain only one archaeal histone and, by heterologous expression of TK1413 and TK2289, preparations of recombinant HTkA and HTkB homodimers were obtained. Direct comparisons could therefore be made of the locations of archaeal nucleosomes assembled by only HTkA or HTkB, in vivo and in vitro, on *T. kodakarensis* genomic DNA. The archaeal nucleosome profiles were very similar in vivo and in vitro throughout the length of the *T. kodakarensis* genome (Figure 4a). The pattern of nucleosome depletion immediately upstream of translation codons was conserved (Figure 4b) and there was only minimal archaeal nucleosome assembly on the rDNA operon both in vivo and in vitro (Figure 5).

As established for the parental strain (Figure 1a), the ~60 bp fragments of the *T. kodakarensis* genome protected from MN digestion by only HTkA or HTkB assembly in vivo and in vitro also contained 10 bp helical-periodicity repeats of AA/AT/TA/TT and GG/GC/CG/CC dinucleotides, offset by 5 bp, and pentamers containing only A and/or T were under-represented, and those containing only G and/or C were over-represented, relative to their occurrences in the *T. kodakarensis* genome (Additional file 1: Figure S1). Together these results confirm that the positions at which HTkA and HTkB assemble to form archaeal nucleosomes are predominantly determined by the *T. kodakarensis* genome sequence and, as concluded from eukaryotic nucleosome studies [7,33,38,43-46], from an archaeal genome sequence [39], it should be possible to predict where archaeal nucleosomes will preferentially assemble in vivo.

### Evolutionary conservation of the nucleosome positioning code

**Figure 6:**
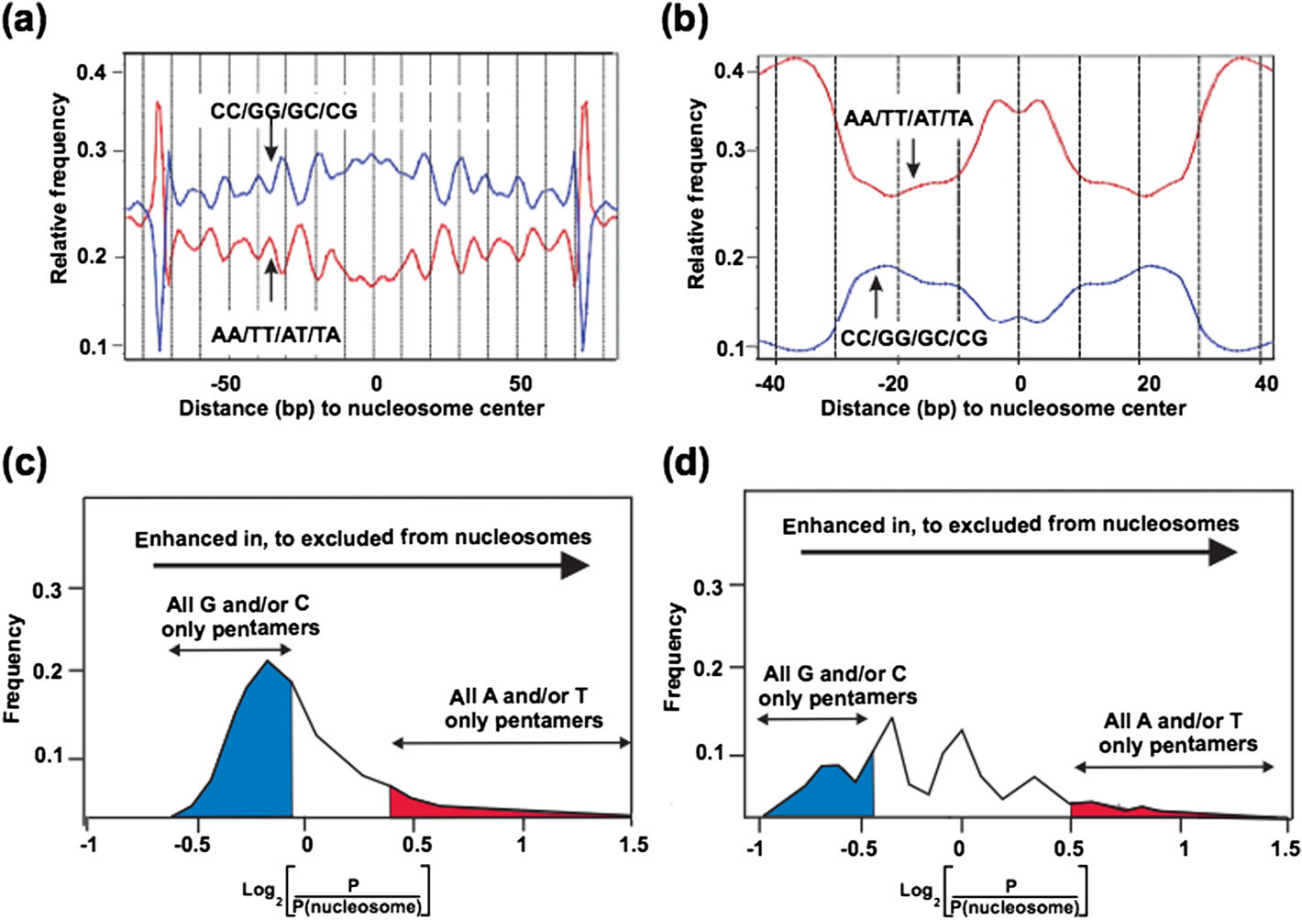
**Archaeal and eukaryotic nucleosomes are positioned by conserved sequences.** The frequencies of occurrence of AA/AT/TA/TT and CC/GG/GC/CG dinucleotides, at each position relative to the center of **(a)** eukaryotic nucleosomes assembled by chicken histones on *T. kodakarensis* genomic DNA, and **(b)** of archaeal nucleosomes assembled by *T. kodakarensis* histones on yeast genomic DNA. **(c)** The ratio of the presence and absence of all pentamers in the *T. kodakarensis* DNA protected from MN digestion by assembly into eukaryotic nucleosomes, and **(d)** in yeast DNA assembled into archaeal nucleosomes. As indicated, all pentamers that contained only G and/or C were preferentially incorporated into both eukaryotic and archaeal nucleosomes (blue regions) whereas all pentamers that contained only A and/or T (red region) were preferentially excluded nucleosomes.

source (eukaryotic *versus* archaeal), we assembled nucleosomes using eukaryotic histones with archaeal DNA and archaeal histones with eukaryotic DNA. As expected, based on many previous studies, chicken histone octamers bound *M. thermoautotrophicus* and *T. kodakarensis* genomic DNAs into nucleosomes that protected ~147 bp DNA fragments from MN digestion and, as in Figure 1a, ~60 bp fragments of yeast genomic DNA were protected from MN digestion by archaeal histone assembly. Sequencing these fragments generated 2 to 5 million unique reads, equating to ~60- to 270-fold coverage per bp, per 60 bp or 147 bp fragment. Analyses of the sequences confirmed that the presence of oscillating dinucleotide repeat patterns and the exclusion of A/T-rich pentamers in all the nucleosome-incorporated DNAs (Figure 6). Aligning the nucleosome profiles confirmed that most sites at which archaeal nucleosomes assembled in vivo were sites at which eukaryotic nucleosomes also assembled preferentially, although less abundantly, in vitro. Chicken histone octamers often formed nucleosomes that encompassed two adjacent sites of preferred archaeal nucleosome assembly (Additional file 2: Figure S2).

### Archaeal histone deletion changes transcription and nucleosome positioning

**Figure 7:**
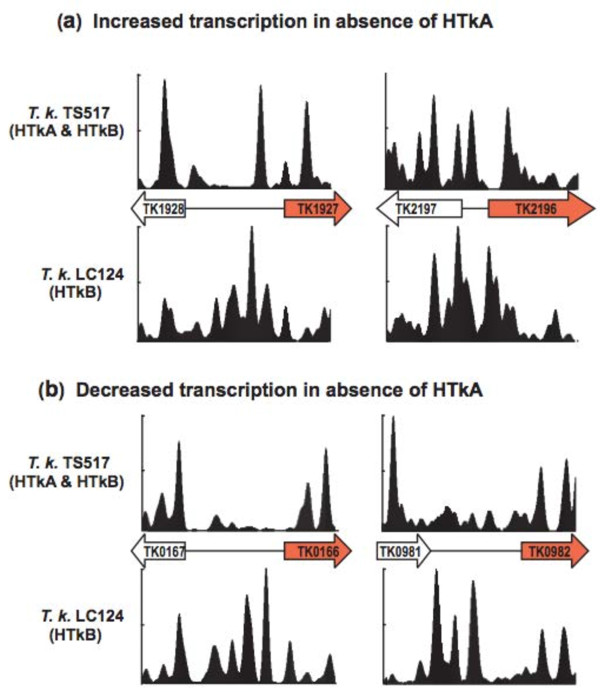
**Nucleosome profiles, related to transcription changes that result from archaeal histone deletion.** The profiles of nucleosomes assembled in vivo in *T. kodakarensis* TS517 and LC124 are respectively shown above and below the 1-kbp genomic region of interest [34,39]. Intergenic regions are depicted as a single line and the genes that **(a)** had increased transcription or **(b)** had decreased transcription in the absence of HTkA [13] are shaded red.

*T. kodakarensis* LC124 (ΔTK1413) and LC125 (ΔTK2289) exhibit no detectable growth defects but microarray hybridizations revealed that transcripts of 3% to 4% of genes increased or decreased in abundance when compared with their abundances in the parental strain *T. kodakarensis* TS517 [13]. Comparing the nucleosome profiles in the intergenic regions immediately upstream of these genes in *T. kodakarensis* TS517 versus LC124 or LC125 revealed clear differences, but no consistent pattern that correlated directly with an increase or decrease in transcript abundance. For example, in *T. kodakarensis* LC124 that lacks HTkA, transcripts of TK2196 and TK1927 increased in abundance 3.1- and 3.4-fold, respectively, but although there was decreased nucleosome assembly upstream of TK2196, there was increased assembly upstream of TK1927 (Figure 7a). Similarly, in the absence of HTkA, transcripts of TK0166 and TK0982 decreased 5.3- and 3.5-fold, respectively, and there was increased nucleosome assembly upstream of TK0166, but there were both regions with decreased and increased nucleosome assembly upstream of TK0982 (Figure 7b).

## Discussion

### Conservation and ancestral origin of the nucleosome positioning code

Whether a sequence will favor or disfavor assemble into a nucleosome can be predicted from the below- or above-average energy requirements needed to distort that sequence into the circular configuration of DNA wrapped around the nucleosome core [19,47,48]. To a large extent, these predictions have been confirmed experimentally by the sequences of DNA isolated from eukaryotic nucleosomes and so in the definition of a nucleosome positioning code [5-8,19,21,33,38,43]. The results reported here confirm that two basic features of this code, namely a 10 bp periodicity of AA/AT/TA/TT dinucleotides offset by 5 bp from GG/GC/CG/CC dinucleotides, and the exclusion of A/T-rich oligonucleotides also direct archaeal nucleosome assembly in vivo and in vitro. In the structures established for the eukaryotic nucleosome [49,50], the AA/AT/TA/TT dinucleotide repeats facilitate DNA wrapping as the dinucleotides that most readily accept the distortion needed [48], where the minor groove faces inwards towards the histone octamer. The GG/GC/CG/CC dinucleotide repeats, offset by half a helical turn, facilitate the distortion needed at each site where the minor groove faces outwards and so away from the nucleosome core [3,49,50]. To date, despite a significant effort, there is no high resolution structure of an archaeal nucleosome available but, given that the archaeal and eukaryotic histone folds are virtually identical [9,51] and that DNA-histone interactions are almost entirely mediated by histone fold residues [52], it seems highly likely that the same wrapping mechanism is employed and so DNA distortion is required to assemble archaeal nucleosomes. Given this conservation, the nucleosome positioning code almost certainly predates the Eukaryotes, and co-evolved with histones and the histone-fold based mechanism of DNA compaction early in the archaeal lineage, before the divergence of *Euryarchaea*, *Nanoarchaea* and *Thaumarchaea*[9-11].

### Archaeal nucleosome positioning and gene expression

As in *Bacteria*, many archaeal genes are organized into operons and so are co-transcribed from a single promoter and, in the absence of a nuclear membrane; transcription and translation are coupled [53]. However, archaeal promoters and the transcription machinery are substantially more similar to their eukaryotic than bacterial counterparts. Archaeal transcription initiation requires that both transcription factors and RNA polymerase be recruited to the promoter [35] and archaeal histone binding to promoter regions has been shown, alternatively, to inhibit or to stimulate transcription in vitro [54,55]. Without a transcriptome, we chose to determine the locations at which HTkA and HTkB assembled into nucleosomes on *T. kodakarensis* genomic DNA, both in vivo and in vitro, relative to translation start codons. This is a reasonable proxy for transcription initiation sites as most archaeal promoters are located within short intergenic regions and so are close to a start codon. As the results revealed, on a genome wide basis, archaeal nucleosomes are excluded from the DNA immediately upstream of open reading frames, and these nucleosome depleted regions (NDRs) are flanked by regions with above average nucleosome occupancy (Figure 4b). This is very similar to the pattern of nucleosome occupancy established in promoter regions and around the downstream transcription start sites in the yeast genome [1,3,26,56]. The conservation of this nucleosome organization argues strongly for a participatory role in gene expression. Possibly, avoiding nucleosome assembly in the promoter region coupled with the presence of a nucleosome at the transcription start site evolved as a generic system to facilitate pre-transcription complex assembly while preventing immediate transcription initiation. If so, a mechanism would then also be needed to remove the inhibitory nucleosome when transcription should occur. In Eukaryotes, many complexes have evolved that catalyze histone modifications, nucleosome remodeling, repositioning and/or eviction that relieve transcription inhibition [3] but, to date, there is no evidence for archaeal histone modifications nor for archaeal nucleosome remodeling complexes.

Consistent with the presence of an archaeal nucleosome impeding transcription [54], presumably to maximize rRNA synthesis, archaeal nucleosome assembly was strongly deterred by the rDNA operon sequences, in vivo and in vitro, in both *T. kodakarensis* (Figure 6) and in *M. thermautotrophicus* (Additional file 3: Figure S3). To test the prediction that the presence of an archaeal nucleosome in a promoter region was sufficient to prevent downstream transcription, we compared the archaeal nucleosomes profiles upstream of genes whose transcription was known to increase or decrease in response to the absence of HTkA or HTkB [13]. There were substantial differences in these profiles, in both the abundance and positioning of archaeal nucleosomes, when compared with the parental strain with both histones (Figure 7) but there was no consistent correlation. An increase or decrease in transcription did not simply result from the absence or presence of an archaeal nucleosome in a promoter region.

### Supporting report

The results of a similar and complementary investigation were published [57] while this report was being finalized. Ammar et al. [57] determined the locations of archaeal nucleosomes assembled in vivo in *Haloferax volcanii*, also a euryarchaeon, but a species with a relatively high 65% G + C content, and one with a single atypical archaeal histone that has two histone folds fused into one polypeptide [9,15,58]. Both positioning investigations established that G/C-rich sequences predominate at the center of an archaeal nucleosome but a 10 bp periodicity of AA/AT/TA/TT dinucleotides was not detected in the DNA incorporated into nucleosomes in *Hlf. volcanii*. In both studies, a NDR flanked by increased nucleosome assembly was documented in intergenic regions but, with a transcriptome available, Ammar et al. [57] were able to define the location of the NDR relative to the sites of transcription initiation rather than, as here, to translation start codons. They did not report confirmatory in vitro positioning studies, but the nucleosome positioning in vivo in *Hlf. volcanii* was, as established here both in vivo and in vitro, almost certainly directed primarily by the *Hlf. volcanii* genome sequence.

## Conclusions

The results reported establish that an archaeal histone and genome sequence are sufficient for positioned archaeal nucleosome assembly. They confirm that the primary sequence motifs known to facilitate and direct histone assembly into eukaryotic nucleosomes [5-8] also direct nucleosome assembly in Archaea and that this positioning mechanism therefore almost certainly originated in a common ancestor of *Archaea* and Eukaryotes. DNA compaction is often described as the primary function of nucleosomes but positioned nucleosomes clearly also participate in regulating eukaryotic gene expression [3,59,60] and nucleosome positioning, as a regulatory mechanism, likely predates nucleosome assembly for DNA compaction and archiving. When compared with eukaryotic genomes, archaeal (and bacterial) genomes are very small, and many different proteins have been described that participate both in gene expression and prokaryotic genome compaction [15]. The first histone may have evolved as a protein that bound preferentially to sequences that encoded amphipathic peptide helices [61] with this sequence-directed binding participating in gene regulation. But, given that the histone fold mechanism of DNA binding results in DNA wrapping, this would have also inherently resulted in DNA compaction. When evaluated in terms of the length of DNA compacted per unit of protein, histone wrapping is very efficient, and it is possible that it was the availability of this mechanism, employed by all Eukaryotes, that allowed the massive genome expansion needed for eukaryotic evolution [62]. It remains to be determined if archaeal histones still function primarily as regulators, as suggested by their depletion in intergenic regions [57], or if genome compaction is their primary function consistent with their observed assembly here throughout the full length of the *T. kodakarensis* and *M. thermautotrophicus* genomes.

## Methods

### Isolation of genomic DNA and archaeal nucleosomes assembled in vivo

Cells from exponentially growing cultures of *M. thermautotrophicus*, *T. kodakarensis* TS517 (Δ*pyrF;* Δ *trpE::pyrF;* Δ *TK0664*), LC124 (Δ *pyrF;* Δ*trpE::pyrF;* Δ *TK0664;* Δ TK1413) and LC125 (Δ *pyrF;* Δ *trpE::pyrF;* Δ*TK0664;* ΔTK2289) [13] were harvested by centrifugation, flash frozen and genomic DNA preparations isolated from aliquots of these cells as previously described [36,40]. The remainder were ruptured by grinding in frozen micrococcal nuclease (MN) buffer [50 mM Tris (pH 8), 1 mM CaCl_2_, 100 mM NaCl], and the lysates allowed to thaw at 4°C. Aliquots were incubated with MN (1 U/μl) at 37°C, and the nuclease digestion then terminated, after increasing periods of digestion, by addition of 250 mM EDTA, 1% SDS, 200 mM NaCl. Following incubation with RNase A (10 mg/ml) for 60 min at 42°C, the DNA molecules that remained were purified by phenol:chloroform extraction, concentrated by ethanol precipitation, and separated by electrophoresis through 3.5% NuSieve agarose gels (Fisher Molecular Biology, Trevose, PA) or 6% polyacrylamide gels. Gel fragments that contained DNA molecules with ~60 bp lengths were excised, crushed and the DNA molecules eluted by incubation overnight at 37°C in 300 mM sodium acetate, 1 mM EDTA (pH 8), 0.1% SDS. The DNA molecules were concentrated by ethanol precipitation, and prepared for sequencing (see below).

### Archaeal histone gene cloning, expression and purification of recombinant HTkA and HTkB

The genes, TK1413 and TK2289, that encode HTkA and HTkB respectively in *T. kodakarensis* TS517 [34], were PCR-amplified and cloned into plasmid pQE-80 (Qiagen, Valencia, CA) generating plasmids pTS600 (TK1413) and pTS601 (TK2289) that were transformed into *Escherichia coli* Rosetta 2 (EMD-Millipore, Billerica, MA). Cultures of the transformants were grown to the late exponential phase in LB medium that contained 100 μg ampicillin/ml and 30 μg chloramphenicol/ml at 37°C, and recombinant HTkA or HTkB synthesis was then induced by adding isopropyl β-D-1-thiogalacto-pyranoside (500 μM final concentration) and continued incubation for 3 h at 37°C. The cells were harvested by centrifugation, resuspended (0.33 g wet cell pellet/ml) in 25 mM Tris–HCl (pH 7), 0.1 mM EDTA, 50 mM NaCl, lysozyme (100 μg/ml) added and the mixtures held ice for 30 min. Phenylmethanesulfonyl fluoride (Sigma, St. Louis, MO) was added (100 μg/ml) and cells were ruptured by repeated passage through a French press. The lysates were clarified by centrifugation at 4°C (60,000 g, 20 min), MgCl_2_ (5 mM) and DNase I (40 μg/ml) added, the mixtures incubated for 1 h at 37°C and then at 85°C for 20 min. Following further centrifugation (60,000 g, 30 min, 4°C), the supernatants generated were loaded onto 5 ml Hi-Trap heparin columns (GE Healthcare; Pataskala, OH). Recombinant HTkA and HTkB were eluted by passage of 10 column volumes of linear 50 to 500 mM, and 200 to 700 mM gradients of NaCl, respectively, dissolved in 25 mM Tris–HCl (pH 7). The eluate fractions that contained HTkA or HTkB were identified by Commassie-staining of the proteins in samples of the fractions separated by electrophoresis through 22% (w/v) denaturing polyacrylamide gels. These fractions were combined and the protein solution concentrated (final volume of ~0.5 ml) by centrifugation through a pre-rinsed Vivaspin 6 centrifugal concentrator (5 K molecular weight cut off; Sartorious AG, Bohemia, NY). The solutions were adjusted to contain 600 mM NaCl in 25 mM Tris–HCl (pH 7) and then passaged through Sephacryl S-100 HR 16/40 column (GE Healthcare) at a flow rate of 0.5 ml/min. Fractions that contained HTkA or HTkB, identified by Commassie-blue staining after electrophoresis of aliquots through 22% denaturing polyacrylamide gels, were pooled and concentrated (final volume of ~2 ml) by centrifugation again through pre-rinsed Vivaspin 6 centrifugal concentrators (5 K molecular weight cut off). These proteins solution, >99% purified archaeal histone, were dialyzed against in 25 mM Tris–HCl (pH 7), 500 mM NaCl, 50% (v/v) glycerol, and stored at −20°C.

### Purification of eukaryotic histones

Chicken histone octamers were purified from erythrocytes by salt extraction and by hydroxyapatite column chromatography as previously described [63].

### Archaeal and eukaryotic nucleosome assembly *in vitro*

Eukaryotic nucleosomes were assembled in vitro by salt dialysis in 200 μl reaction mixtures that contained 50 μg of genomic DNA and 30 μg of chicken histone octamers [6]. Archaeal nucleosomes were reconstituted by mixing 50 μg of genomic DNA with 30 μg archaeal histone tetramers. The complexes formed were dialyzed into MN digestion buffer, and aliquots containing ~2.5 μg of DNA were incubated with 0.1 U MN/ μl for 5 min at 37°C. The MN digestions were stopped by addition of 125 mM EDTA, 200 mM NaCl, and the DNA molecules remaining were isolated by phenol:chloroform extraction, concentrated by ethanol precipitation and separated by electrophoresis through 6% polyacrylamide or 3.5% NuSieve agarose gels. Gel fragments that contained the ~60 bp, or ~147 bp, DNA molecules protected from MN digestion by incorporation into archaeal or eukaryotic nucleosomes, respectively, were excised and the DNA molecules extracted, purified and prepared for DNA sequencing as described above.

### ABI SOLiD sequencing of DNA fragments

The ends of the ~60 bp and ~147 bp DNA fragments were repaired and 5’-phosphorylated by incubation in DNATerminator end-repair kits, as recommended by the manufacturer (Lucigen Corp., Middleton, WI). SOLiD adapters were ligated and the DNA molecules PCR amplified (very low cycle number) and sequenced by using the Applied Biosystems protocol for SOLiD fragment paired-end sequencing [64]. Sequencing generated from 2 to 12 million unique reads which, depending on the experiment, equated to 60- to 800-fold coverage per 60 bp or 147 bp nucleosome footprint.

### Analysis of DNA reads generated by pair-end sequencing

We first selected reads of length 55–65 bp (nucleosome of 60 bp lengths) to construct the center-weighted nucleosome occupancy scores. If a read length was odd, a Gaussian weight of *exp*(−0.5 * (*d*/10)^2^) was assigned to a position *d* bp away from the center of the read for *d* ≤ 25. If a read length was even, then positions *i* − 1 and *i* were treated as the possible nucleosome centers. For example, for a 60 nucleotide sequence *i* = 31, and so the two potential centers were at positions 30 and 31. Each center in an even read was, in turn, assigned a weight of 0.5 * *exp*(−0.5 * (*d*/10)^2^) for a position *d* bp away from the center and the values for both positions were then divided by 2. The center-weighted occupancy score for any given position was defined as the aggregation of the weighted scores from all reads. We identified well-defined peaks on the reads occupancy-curve as putative nucleosome centers by controlling the peak height and steepness simultaneously. To generate AA/AT/TA/TT frequency plots, after defining the nucleosome center positions based on the peaks of center-weighted occupancy score, dinucleotide frequency scores were computed as described by Segal *et al.*[5]. We searched for a sequencing tag of length 60 bp nearest to the peak position in the +/−5 bp region. If no such read existed, we further searched for reads of lengths 61, 59, 62 and 58 bp sequentially within +/− 5 bp region of the peak until the first read was identified. The center of the identified read was treated as the nucleosome center to generate the AA/TT/TA/AT frequency plot. If no such read was identified in the +/−5 bp region, the peak position was treated as the true nucleosome center to generate the alignment. For paired-end MNase sequencing data for 147 bp long nucleosomes, read lengths of 137–157 bp were used. We followed a similar approach as described above and also employed by Brogaard et al. [21,65] to identify the nucleosome centers.

### Analyses of the DNA reads generated by single-end sequencing

For the single-end reads with known start position on the Watson strand, their end positions are unknown. However, since the DNA inserts are mapping nucleosomes, their length must be subject to the constraint of being around one nucleosome repeat length. Thus, if we observe a single-end read on the Watson strand at position *i*, we could practically assume that its end position should be within a region, say [*i* + *a*, *i* + *b*], and follows some distribution. For practical purpose, we let *a* = 51, *b* = 68. We further assumed that the start and end positions of the DNA inserts are independently distributed around the two edges of the nucleosome they map. Let *c*_*i* + 51_, …, *c*_*i* + 68_ be the Crick strand tag numbers in this region. Then the relative frequency defined as $c_{i+k}/\sum_{j=51}^{68}c_{i+j}$ can be used to estimate the probability of a DNA insert ending at position *i* + *k* for *k* = 51, …, 68. Thus, if we observe *w*_*i*_ single-end tags at position *i* from the Watson strand, then we could regard that we had observed *w*_*i*_ paired-end tags ending at *i* + *k* for *k* = 51, …, 68 with respective frequency $w_{i}c_{i+k}/\sum_{j=51}^{68}c_{i+j}$. Likewise, if we observe *c*_*i*_ single-end tags at position *i* from the Crick strand, we would regard that there were *c*_*i*_ paired-end tags ending at *i* − *k* for *k* = 51, …, 68 with respective frequency $c_{i}w_{i-k}/\sum_{j=51}^{68}w_{i-j}$. By this calculation the observed data with $\sum_{i}(w_{i}+c_{i})$ single-end tags are converted approximately to a pseudo data set consisting of $\sum_{i}(w_{i}+c_{i})$ paired-end tags. The approach defined above for paired-end data was then used to define the center-weighted reads occupancy score and the nucleosome centers.

### Primary data

The sequences obtained and detailed descriptions of the computational analyses are available [66]. The *M. thermoautotrophicus* and *T. kodakarensis* genome coordinates and RefSeq transcript annotations used were from the *methTher1*[67] and *therkoda1*[42] genome assemblies available on the Archaeal Genome Browser web site [39].

## Competing interests

The authors declare that they have no competing interests.

## Authors’ contributions

Designed and directed the project: TJS, JNR, YNF-M. Performed experiments: NN, SB, TJS, YNF-M. Analyzed the data: LX, WJ-P, TJS, JNR, YNF-M. Wrote the paper: NN, SB, WJ-P, TJS, JNR, YNF-M. All authors read and approved the final manuscript.

## Supplementary Material

Additional file 1: Figure S1 Shows data that document that archaeal nucleosomes assembled by HTkA and HTkB, in vivo and in vitro, contain 5 bp offset helical repeats of AA/AT/TA/TT and CC/GG/GC/CG dinucleotides and preferentially exclude oligo A/T-rich sequences. Click here for file

Additional file 2: Figure S2 Documents the conserved positioning of archaeal and eukaryotic histone assembly into nucleosomes on *Methanothermobacter thermautotrophicus* genomic DNA. Click here for file

Additional file 3: Figure S3 Documents the absence of archaeal nucleosome assembly in vivo and in vitro on the two rDNA operons present in the *Methanothermobacter thermautotrophicus* genome. Click here for file
