# Supplementary material for: Archaeal nucleosome positioning in vivo and in vitro is directed by primary sequence motifs
Source: BMC Genomics. 2013 Jun 10;14:391. doi: 10.1186/1471-2164-14-391 (PMC3691661; doi:10.1186/1471-2164-14-391)
Supplement: Additional file 1: Figure S1 — Shows data that document that archaeal nucleosomes assembled by HTkA and HTkB, in vivo and in vitro, contain 5 bp offset helical repeats of AA/AT/TA/TT and CC/GG/GC/CG dinucleotides and preferentially exclude oligo A/T-rich sequences. [file 1471-2164-14-391-S1.pdf]

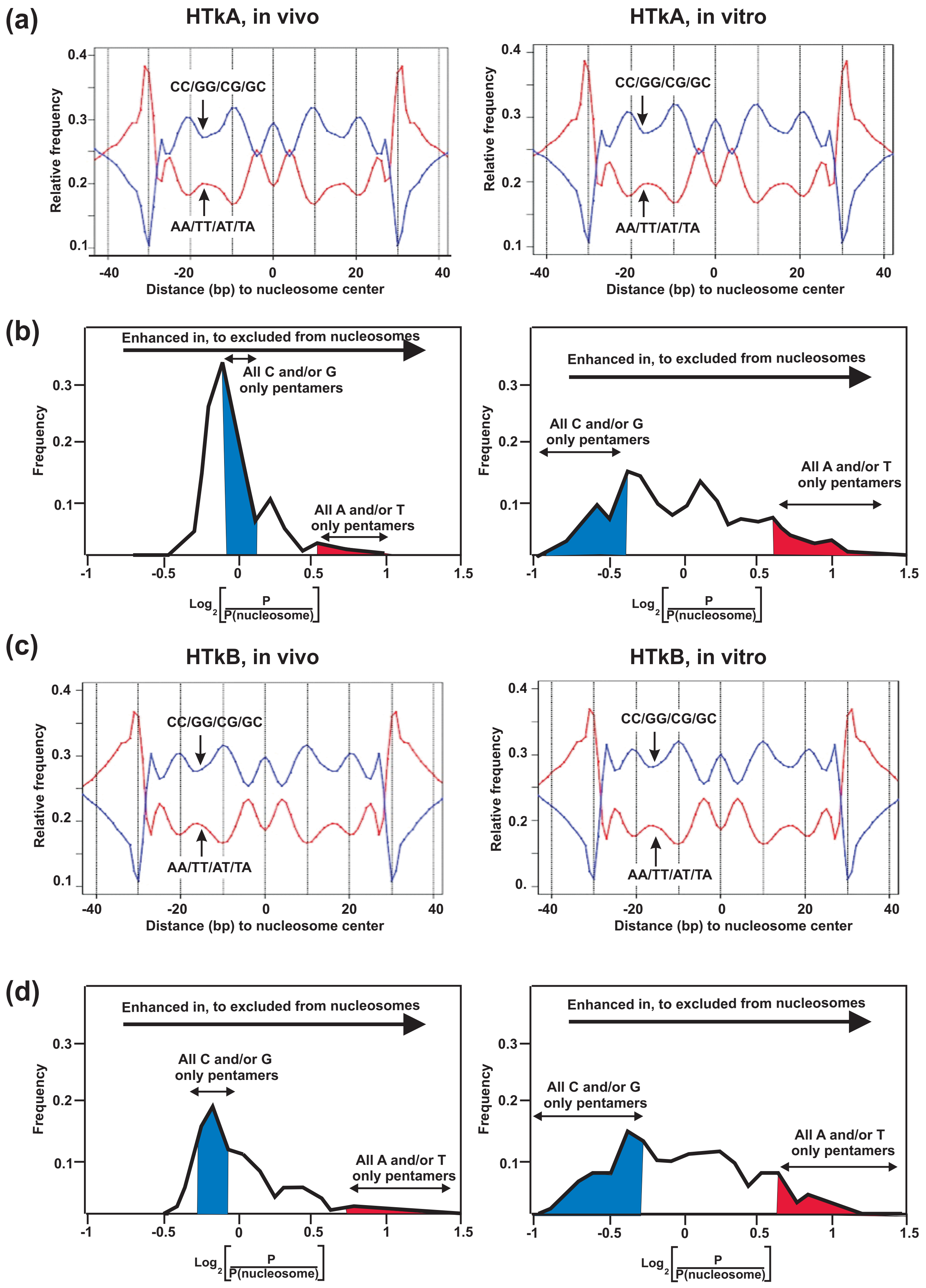
(a)
HTkA, in vivo
HTkA, in vitro
CC/GG/CG/GC
AA/TT/AT/TA
Relative frequency
Distance (bp) to nucleosome center
(b)
Enhanced in, to excluded from nucleosomes
All C and/or G only pentamers
All A and/or T only pentamers
Frequency
Log2 [P / P(nucleosome)]
(c)
HTkB, in vivo
HTkB, in vitro
CC/GG/CG/GC
AA/TT/AT/TA
Relative frequency
Distance (bp) to nucleosome center
(d)
Enhanced in, to excluded from nucleosomes
All C and/or G only pentamers
All A and/or T only pentamers
Frequency
Log2 [P / P(nucleosome)]

Figure S1

Legend: Supplementary Figure S1

Archaeal nucleosomes assembled by HTkA and HTkB, in vivo and in vitro, contain 5 bp offset helical repeats of AA/AT/TA/TT and CC/GG/GC/CG dinuculeotides and preferentially exclude oligoA/T-rich sequences. The frequencies of occurrence of AA/AT/TA/TT and CC/GG/GC/CG dinucleotides, at each position relative to the center of an archaeal nucleosome assembled on *T. kodakarensis* genomic DNA by **(a)** HTkA in vivo and in vitro, and **(c)** by HTkB in vivo and in vitro. The ratios of the presence and absence of all pentamers protected from MN digestion by **(b)** HTkA nucleosome assembly in vivo and in vitro, and **(d)** by HTkB nucleosome assembly in vivo and in vitro. As noted, pentamers that contained only G and/or C were incorporated preferentially by both HTkA and HTkB into archaeal nucleosomes in vivo and in vitro (blue regions), whereas pentamers that contained only A and/orT (red regions) were preferentially excluded from nucleosome assembly both in vivo and in vitro.
