# Supplementary material for: Archaeal nucleosome positioning in vivo and in vitro is directed by primary sequence motifs
Source: BMC Genomics. 2013 Jun 10;14:391. doi: 10.1186/1471-2164-14-391 (PMC3691661; doi:10.1186/1471-2164-14-391)
Supplement: Additional file 2: Figure S2 — Documents the conserved positioning of archaeal and eukaryotic histone assembly into nucleosomes on Methanothermobacter thermautotrophicus genomic DNA. [file 1471-2164-14-391-S2.pdf]

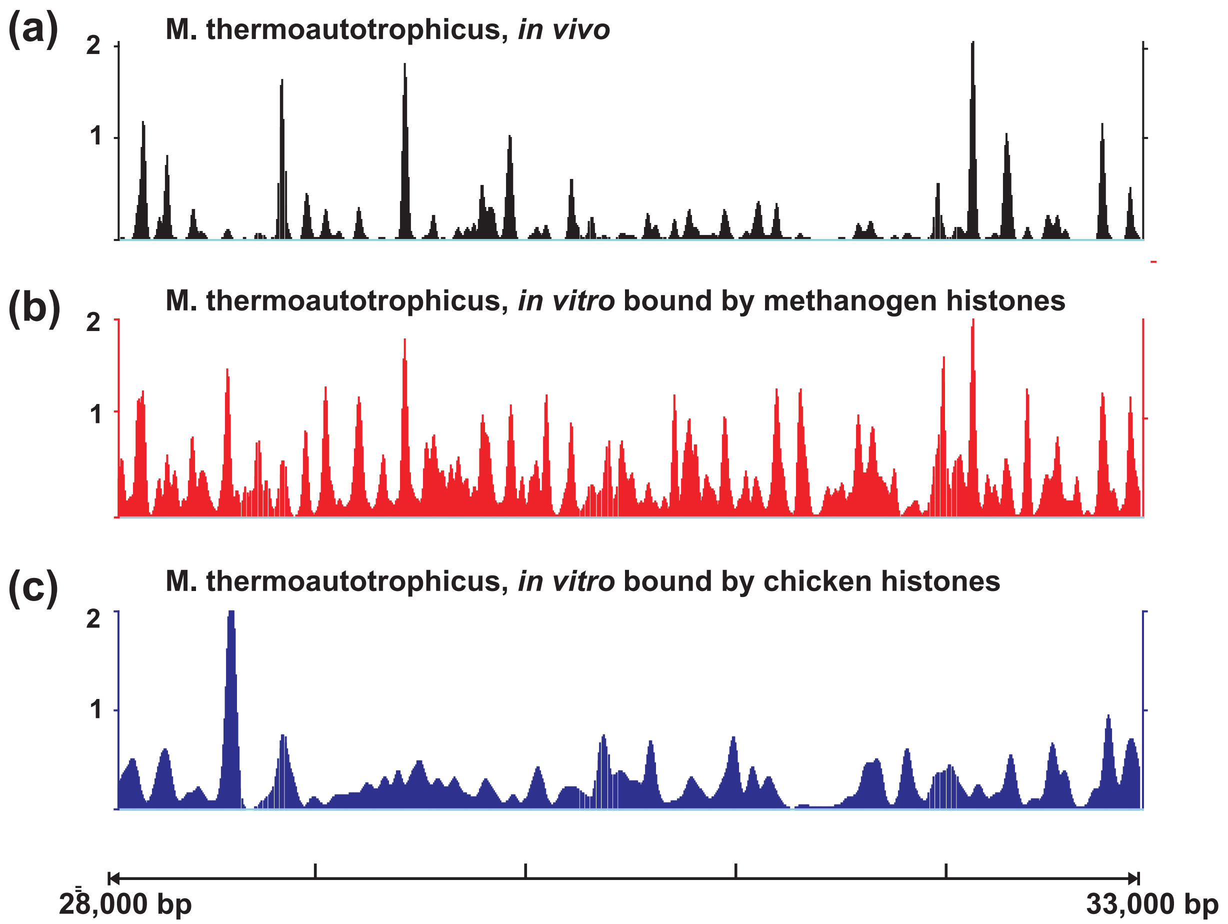
(a)
M. thermoautotrophicus, *in vivo*
2
1
(b)
M. thermoautotrophicus, *in vitro* bound by methanogen histones
2
1
(c)
M. thermoautotrophicus, *in vitro* bound by chicken histones
2
1
28,000 bp
33,000 bp

Figure S2

Legend: Supplementary Figure S2

Conservation of archaeal and eukaryotic nucleosome profiles. The profiles of nucleosomes assembled on a 5 Kbp region of the *M. thermautotrophicus* genome (nucleotides 28,000 to 33,000 in the genome annotation [http://archaea.ucsc.edu] **(a)** in vivo, **(b)** in vitro by methanogen histones, and **(c)** in vitro by chicken histones.
