# Supplementary material for: Archaeal nucleosome positioning in vivo and in vitro is directed by primary sequence motifs
Source: BMC Genomics. 2013 Jun 10;14:391. doi: 10.1186/1471-2164-14-391 (PMC3691661; doi:10.1186/1471-2164-14-391)
Supplement: Additional file 3: Figure S3 — Documents the absence of archaeal nucleosome assembly in vivo and in vitro on the two rDNA operons present in the Methanothermobacter thermautotrophicus genome. [file 1471-2164-14-391-S3.pdf]

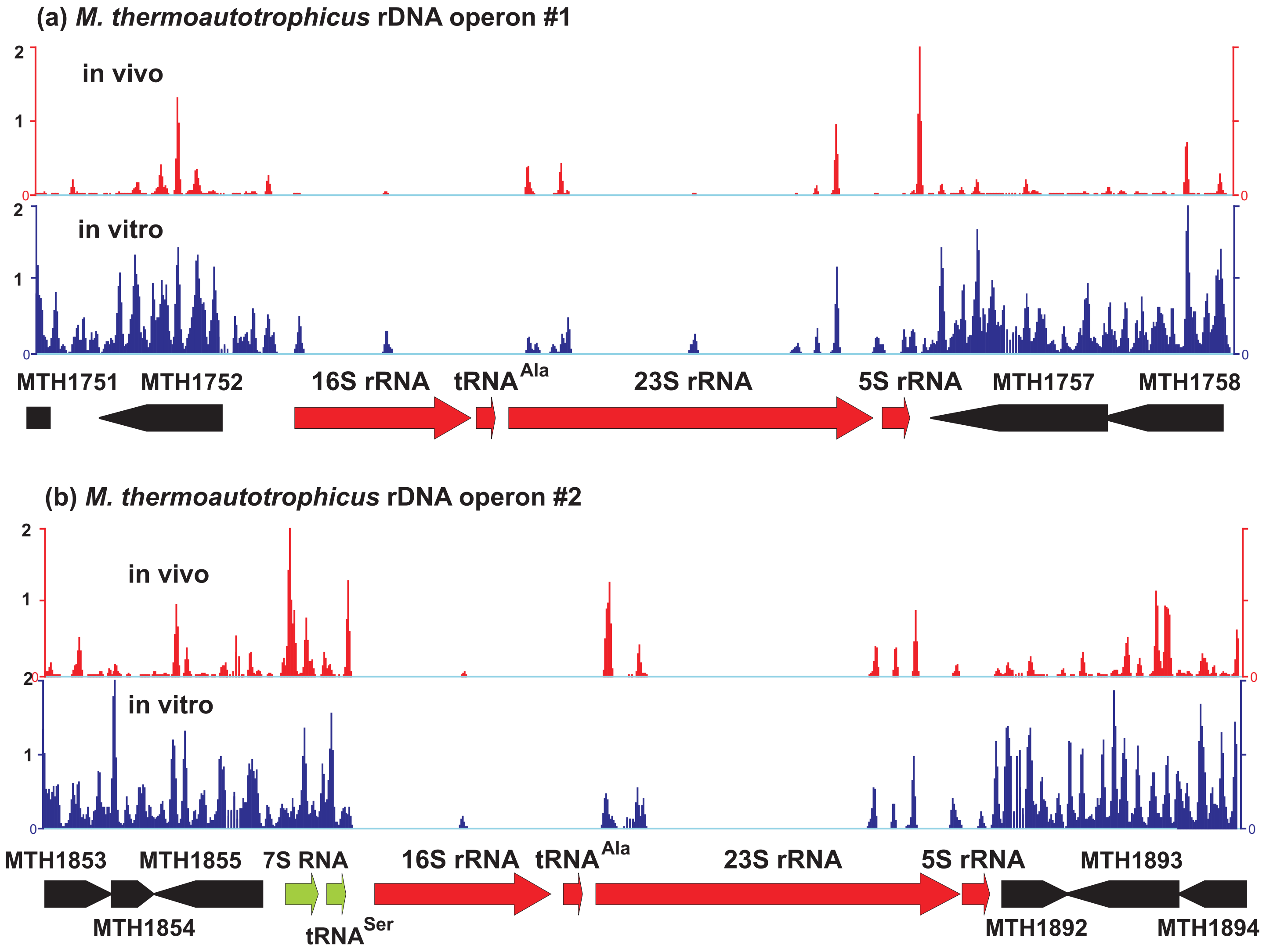

(a) M. thermoautotrophicus rDNA operon #1
in vivo
in vitro
2
1
0
MTH1751
MTH1752
16S rRNA
tRNA Ala
23S rRNA
5S rRNA
MTH1757
MTH1758
(b) M. thermoautotrophicus rDNA operon #2
in vivo
in vitro
MTH1853
MTH1855
7S RNA
MTH1854
tRNA Ser
MTH1893
MTH1892
MTH1894

Figure S3

Legend to Supplementary Figure S3.

Profiles of the archaeal nucleosomes assembled by methanogen histones in vivo and in vitro on 10 Kbp regions of the *M. thermautotrophicus* genome [41]. The *M. thermautotrophicus* genome contains two rDNA operons located from genome position 1,602,209 to 1,607,044 (operon #1) and from 1,718,786 to 1,723,863 (operon #2). As illustrated, both operons encode 16S rRNA, $tRNA^{Ala}$, 23S rRNA and 5S rRNA sequences (red arrows), and genes encoding the 7S RNA and a $tRNA^{Ser}$ (green arrows) are located immediately adjacent operon #2.
